# Challenges in the Recognition and Management of Metastatic Sarcomatoid Carcinoma Masquerading As Post-traumatic Hematoma

**DOI:** 10.7759/cureus.87068

**Published:** 2025-06-30

**Authors:** Abigayle Wyer, Mena Louis, Richard Adams, Raven Richardson, Ezra Ellis, Brian Gibson

**Affiliations:** 1 General Surgery, Northeast Georgia Medical Center Gainesville, Gainesville, USA; 2 General Surgery, St. George's University School of Medicine, True Blue, GRD; 3 Pathology, Northeast Georgia Medical Center Gainesville, Gainesville, USA; 4 Trauma and Acute Care Surgery, Northeast Georgia Medical Center Gainesville, Gainesville, USA

**Keywords:** diagnostic challenges, immunohistochemistry, metastatic lung cancer, postoperative management, psychological support, sarcomatoid carcinoma, soft tissue metastasis

## Abstract

Sarcomatoid carcinoma is an aggressive and rare form of cancer characterized by rapid metastatic spread and diagnostic complexity. This case describes a 68-year-old male presenting initially with persistent thigh swelling and pain following minor trauma. His symptoms were accompanied by systemic signs, including night sweats, constipation, abdominal pain, and dark stools. Despite persistent clinical deterioration, initial imaging suggested a benign etiology, such as hematoma or infection, leading to delayed diagnosis. Subsequent CT scans revealed significant intra-abdominal disease involving the bowel and omentum, with metastatic pulmonary involvement identified upon further imaging.

Surgical exploration uncovered extensive metastatic sarcomatoid carcinoma originating from the lung, involving the bowel, omentum, and thigh soft tissues, necessitating extensive resection and complex bowel reconstructions. Immunohistochemical analysis confirmed pulmonary origin, highlighting the critical role of pathology in accurate diagnosis and treatment planning. Postoperative care was complicated by prolonged ileus, aspiration pneumonia, and persistent leukocytosis, requiring intensive multidisciplinary management. The patient’s recovery emphasized the need for aggressive supportive care, early complication management, and psychological support following an unexpected diagnosis of advanced malignancy.

Clinicians should consider metastatic malignancy when evaluating soft tissue lesions unresponsive to conservative management, especially if systemic symptoms are present. Early biopsy and definitive imaging interpretations are crucial for prompt diagnosis and improved outcomes. Comprehensive care strategies, including surgical intervention, multidisciplinary postoperative care, and psychological support, are essential for managing the complex challenges posed by metastatic sarcomatoid carcinoma.

## Introduction

Sarcomatoid carcinoma is an aggressive and poorly differentiated subtype of carcinoma characterized by both epithelial and mesenchymal histologic features [1]. It most commonly originates in the lungs but may also arise from the kidneys, urinary bladder, or other visceral organs [2]. Clinically, sarcomatoid carcinomas tend to present aggressively, often demonstrating rapid local progression and widespread metastatic disease at diagnosis [3]. This tumor type poses significant diagnostic challenges due to its heterogeneous morphology, nonspecific clinical manifestations, and frequent confusion with benign conditions or primary sarcomas [4].

Metastatic sarcomatoid carcinoma typically involves common metastatic sites such as lymph nodes, liver, bones, and brain [5]. However, unusual presentations, including soft-tissue metastases or extensive intra-abdominal involvement, are relatively uncommon and may obscure initial clinical impressions, complicating early diagnosis and intervention [6]. As such, clinicians must maintain a high degree of suspicion, particularly in cases of persistent and progressive soft tissue lesions or unexplained systemic symptoms such as weight loss, night sweats, and gastrointestinal changes [7].

Soft tissue metastases originating from a primary pulmonary sarcomatoid carcinoma are especially rare, often initially misdiagnosed as trauma-induced hematomas or infectious processes [8]. In these instances, the absence of resolution or progression despite conservative therapy or seemingly appropriate antibiotic regimens should prompt clinicians toward aggressive diagnostic interventions [9]. Definitive imaging combined with early biopsy procedures plays a critical role in establishing accurate diagnoses, guiding subsequent multidisciplinary management, and improving patient outcomes. Considering its aggressive biology, early identification and surgical intervention, complemented by a multidisciplinary approach involving oncology, surgery, radiology, and pathology, are essential to optimize patient care. Despite therapeutic advances, the prognosis remains guarded, reinforcing the necessity for prompt diagnosis, effective symptom palliation, and attentive management of complications [10].

## Case presentation

A 68-year-old male with a significant past medical history of coronary artery disease status post two stent placements, asthma, hypertension, cholecystectomy, left inguinal hernia, and an incidental left renal mass presented to the emergency department complaining of left thigh pain and swelling lasting approximately four months. He initially associated the onset of symptoms with minor trauma after striking his leg against a pipe; however, he denied any associated bruising or skin breaks at the injury site. Approximately three months later, persistent swelling and escalating pain in the affected thigh developed, particularly exacerbated by ambulation and the supine position, preventing tolerance of an MRI scan. He additionally reported associated symptoms including constipation, vague abdominal pain, night sweats, and dark, soft stools lasting approximately one month despite self-administering over-the-counter Alka-Seltzer and nightly Tylenol for symptomatic relief.

**Figure 1:**
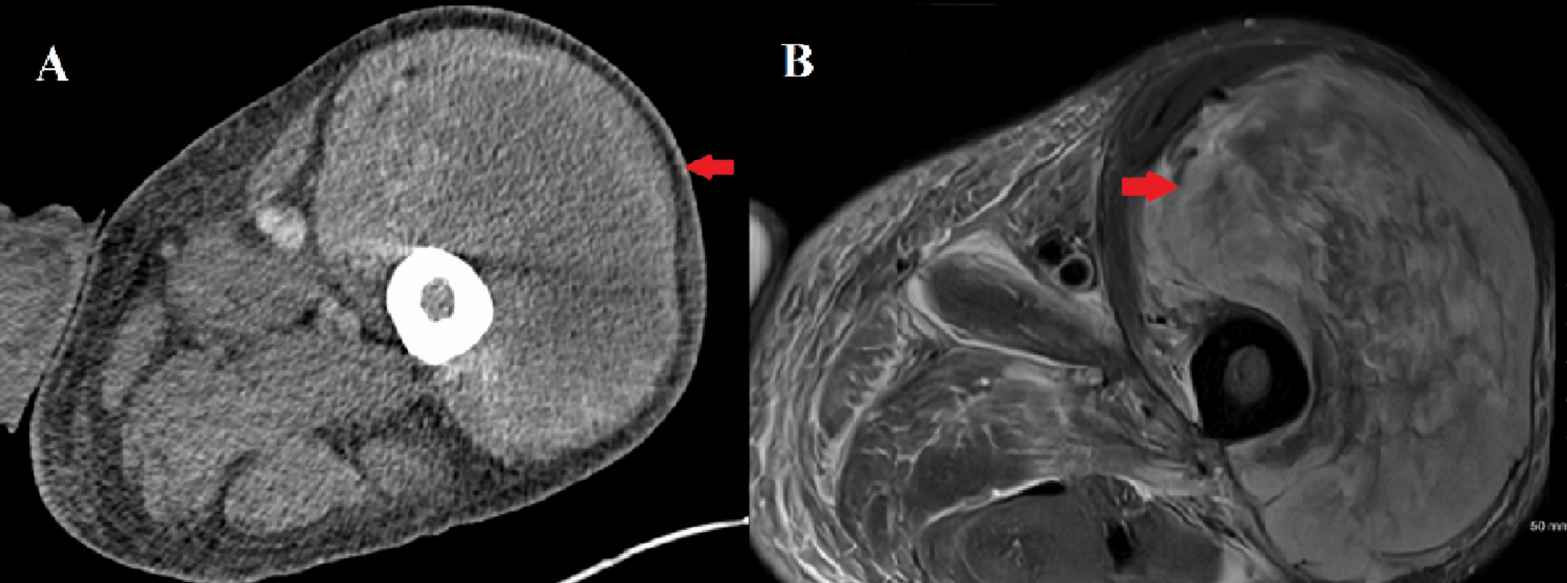
The CT scan and MRI of the patient A: CT scan in axial view reveals a left thigh mass in the anterior compartment measuring 11 x 16 x 11 cm (red arrow); B: MRI in axial view reveals a heterogenous peripherally enhancing mass in the anterior compartment of the thigh, predominantly in the vastus lateralis musculature, measuring 15.2 cm (red arrow)

On initial evaluation, he had a large, painful, swollen left leg mass with no erythema. He did not have abdominal pain on physical exam. Laboratory results revealed marked leukocytosis, mild anemia, hypoalbuminemia, elevated alkaline phosphatase, and a mildly prolonged international normalized ratio (INR) (Table 1). Initial CT imaging demonstrated a sizable heterogenous thigh mass within the anterior muscular compartment measuring 11.0 × 16.3 × 11.3 cm, presumed initially to be a large hematoma (Figure 1, panel A). A subsequent MRI further characterized this lesion as a heterogeneously enhancing mass predominantly involving the vastus lateralis muscle, measuring up to 15.2 cm, again suspected to be a hematoma versus infectious process, with no clear nodularity indicating malignancy (Figure 1, panel B).

**Table 1:** Relevant lab values INR: International normalized ratio

Lab	Value	Reference value
White blood cell (WBC) count	WBC 35.8 × 10³/µL	4,000–11,000 µL
Hemoglobin (Hgb)	12.0 g/dL	12.0 - 15.5 g/dL
Hypoalbuminemia	2.2 g/dL	3.5 to 5.5 g/dL
Alkaline phosphatase	220 U/L	44 and 147 IU/L
INR	1.29	0.8 and 1.1

**Figure 2:**
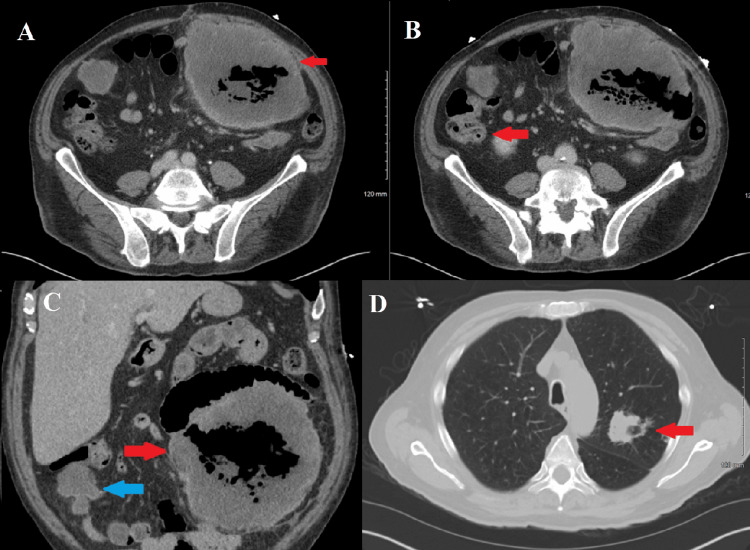
CT scan of the abdomen and chest A: Axial view of abdomen reveals a centrally necrotic peritoneal mass left of the midline measuring 17.5 cm (red arrow); B: Axial view of abdomen reveals a satellite mass in the right abdomen measuring 5 cm (red arrow); C: Coronal view of abdomen reveals a centrally necrotic peritoneal mass left of the midline measuring 17.5 cm (red arrow) and a satellite mass in the right abdomen measuring 5 cm (blue arrow); D: Axial view of chest reveals a mildly spiculated left upper lobe mass (red arrow)

Concurrent CT of the abdomen and pelvis revealed a centrally necrotic peritoneal mass measuring 17.5 cm with satellite lesions, encasing small bowel loops and abutting adjacent bowel with suspicion for transmural invasion, highly suggestive of malignancy (Figure 2, panels A-C). A chest CT demonstrated a mildly spiculated left upper lobe pulmonary mass concerning for malignancy and multiple bilateral pulmonary nodules suggestive of metastatic involvement (Figure 2, panel C). Surgical consultation was recommended given these alarming findings, prompting admission to the trauma and acute care surgery (TACS) service for comprehensive evaluation and intervention.

**Figure 3:**
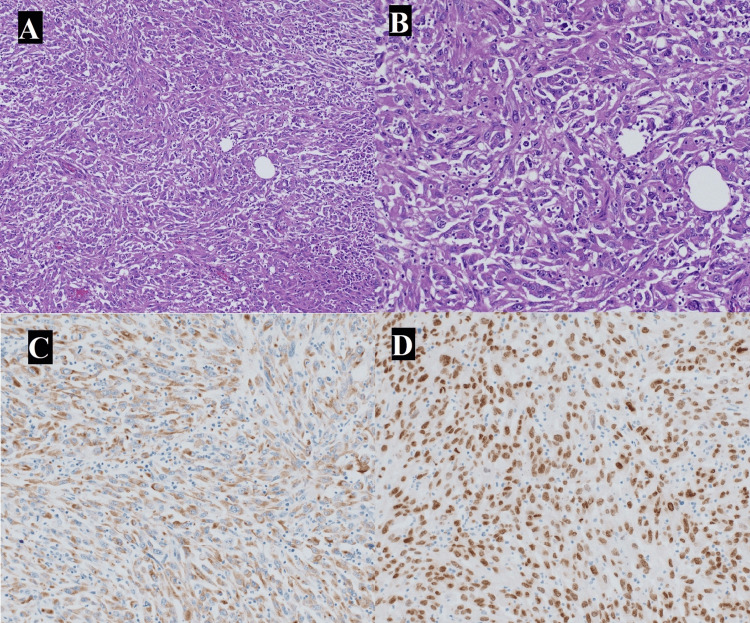
Histopathology images A: Histopathology slide using hematoxylin and eosin stain (H&E) stain demonstrating sarcomatoid carcinoma at 10x magnification; B: Histopathology slide using H&E stain demonstrating sarcomatoid carcinoma at 20x magnification; C: Histopathology slide using pankeratin stain demonstrating sarcomatoid carcinoma; D: Histopathology slide using TTF-1 immunostain demonstrating sarcomatoid carcinoma TTF-1: Thyroid transcription factor 1

The patient underwent exploratory laparotomy, transverse colectomy, small bowel resection, partial omentectomy, peritoneal biopsy, and an incisional biopsy of the left thigh lesion. Intraoperatively, extensive metastatic involvement was noted with a necrotic mass intimately adherent to multiple bowel loops and mesenteric tissues, necessitating substantial resection. Pathological evaluation of the excised tissues revealed metastatic, poorly differentiated sarcomatoid carcinoma with immunohistochemical staining positive for pan-cytokeratin, vimentin, carbonic anhydrase IX (CA-IX), cytokeratin 7 (CK7), and thyroid transcription factor 1 (TTF-1), highly consistent with a pulmonary primary (Figure 3, panels A-D). Postoperative management involved addressing significant ileus and transient aspiration pneumonia. The patient gradually improved clinically and was discharged in stable condition after multidisciplinary care and counseling for his metastatic diagnosis.

## Discussion

Pulmonary sarcomatoid carcinoma (PSC) is a rare and aggressive subtype of non-small cell lung cancer (NSCLC), accounting for approximately 0.1% to 0.4% of all malignant lung tumors [11]. Patients are frequently diagnosed at advanced stages of disease, limiting the feasibility of surgical intervention [12]. Furthermore, PSC is notably resistant to first-line chemotherapy, contributing to its poor prognosis [13]. The overall survival rate for PSC is around 20% lower than that of other NSCLC subtypes, which have a reported survival rate of approximately 45%, with a median survival of only 2.3 months at the time of diagnosis [14]. Although gastrointestinal metastases from lung cancer are relatively uncommon, small bowel involvement has been reported in 2.6% to 10.7% of cases [15]. Those at highest risk include men (73%) with a median age of 63.2 years (range: 36.8-79.7), most of whom are current or former smokers (94.6%) [16].

Metastatic sarcomatoid carcinoma is characterized by rapid growth and unusual metastatic spread, frequently resulting in challenging clinical presentations [17]. In this patient, an initially benign-appearing thigh mass following minor trauma diverted attention from the possibility of metastatic cancer. Persistent swelling, increasing pain, and the presence of systemic symptoms such as night sweats, constipation, abdominal pain, and stool changes were critical indicators that warranted earlier suspicion of a malignant process. Physicians should maintain a high degree of suspicion for metastatic disease when benign explanations like trauma or infection fail to account adequately for progressive clinical deterioration or persistence of symptoms.

Radiologic evaluation posed a significant diagnostic challenge in this patient. Initial CT and MRI scans misinterpreted the thigh mass as a hematoma or potential infection, which delayed biopsy and definitive diagnosis. While MRI is superior for soft tissue evaluation, sarcomatoid carcinomas often contain hemorrhagic and necrotic components that mimic benign lesions radiologically [18]. Thus, reliance on serial imaging without definitive tissue sampling risks significant diagnostic delay. Early biopsy, prompted by lack of improvement or worsening symptoms, is essential for correct diagnosis, timely staging, and appropriate management.

Histopathological confirmation through biopsy remains the cornerstone for diagnosing sarcomatoid carcinoma [1]. Immunohistochemical analysis played a pivotal role in identifying the primary site in this patient [13]. Markers such as pan-cytokeratin, CK7, vimentin, CA-IX, and TTF-1 pointed strongly toward a pulmonary origin, thereby shaping subsequent clinical decisions [19]. Accurate immunohistochemistry is invaluable for treatment planning, prognostication, and targeted therapy selection [20].

The intraoperative management of extensive metastatic disease, as demonstrated in this patient, requires meticulous planning and surgical expertise. Extensive tumor involvement and adherence to multiple loops of bowel necessitated significant resection, including small bowel, colon, and omental segments. Recognizing and effectively managing such widespread metastatic lesions intraoperatively can significantly improve symptoms and quality of life. However, postoperative complications such as prolonged ileus, aspiration pneumonia, and wound management difficulties remain important considerations.

Postoperative management demands careful attention to common complications such as ileus and aspiration pneumonia, both evident in this patient’s course [21]. Aggressive fluid resuscitation, nutritional support, and timely pulmonary interventions mitigate the morbidity associated with extensive surgical resections [22]. Monitoring leukocytosis trends and early management of postoperative infections or aspiration events help prevent severe complications, shorten hospital stays, and improve patient recovery outcomes [23].

Additionally, addressing the psychological impact of a metastatic cancer diagnosis is crucial for comprehensive patient care [24]. The patient's acknowledgment of depressive symptoms highlights the importance of early psychiatric consultation and emotional support. Incorporating mental health assessments into routine oncologic care can significantly improve patient coping strategies, adherence to therapy, and overall quality of life during challenging treatment courses [25].

## Conclusions

Metastatic sarcomatoid carcinoma presenting with atypical soft tissue involvement poses significant diagnostic and therapeutic challenges. Persistent unexplained soft tissue swelling accompanied by systemic symptoms warrants early biopsy, even if imaging suggests benign pathology. Definitive diagnosis via histopathology and immunohistochemistry, aggressive surgical intervention balanced with careful postoperative management, and multidisciplinary supportive care are essential components in managing this aggressive malignancy effectively. Increased clinician awareness of unusual metastatic presentations and a proactive diagnostic approach significantly enhances patient outcomes and quality of care.
